# High dose rate (HDR) brachytherapy quality assurance: a practical guide

**DOI:** 10.2349/biij.2.2.e34

**Published:** 2006-04-01

**Authors:** DA Wilkinson

**Affiliations:** Department of Radiation Oncology, Cleveland Clinic Foundation, Cleveland, United States

**Keywords:** High dose rate brachytherapy, quality assurance

## Abstract

The widespread adoption of high dose rate brachytherapy with its inherent dangers necessitates adoption of appropriate quality assurance measures to minimize risks to both patients and medical staff. This paper is aimed at assisting someone who is establishing a new program or revising one already in place into adhere to the recently issued Nuclear Regulatory Commission (USA) regulations and the guidelines from the American Association of Physicists in Medicine.

## INTRODUCTION

Within five years of its discovery by the Curies in 1898, Ra-226 was being successfully used in brachytherapy [1]. For the next 50 odd years, radium was the isotope of choice for brachytherapy applications, finally yielding to reactor-produced nuclides such as cobalt, cesium, and iridium with much shorter half-lives. These γ-ray emitters, known as “radium substitutes”, were at first used in low dose rate (LDR) implants (< 200 cGy/hr; typically 40 to 80 cGy/hr). More recently, the ability to produce high specific activity Ir-192 sources combined with developments in computer controlled after loader technology has led to widespread adoption of high dose rate (HDR) techniques, i.e. > 1200 cGy/hr [1].

The advantages of HDR treatments include

- greater ease and comfort for the patient (often as an out-patient),
- more precise dose delivery,
- easier dose shaping, and
- less exposure to medical personnel.

However, because of the dangers of using a source with very high activity (10 Ci), it is of utmost importance to have proper quality assurance (QA) procedures in place along with the required dosimetric and planning equipment, and appropriately trained staff. This guide focuses primarily on the first (QA procedures) and the third (training) in this list. It is intended to assist those who are in the process of establishing a program in HDR brachytherapy.

Since each country regulates its own medical use of radioactive material, it is the duty of the medical physicist to establish a quality management program to satisfy those regulations. The focus of this review is regulations of the US Nuclear Regulatory Commission, as contained in 10 CFR Part 10 (medical use of byproduct material) [2] and recommendations made by the American Association of Physicists in Medicine (AAPM). The latter are intended to provide the medical physicist in the USA (and it is hoped other nations as well) the proper guidance to ensure that brachytherapy procedures are carried out safely and with due attention to these rules. There are two AAPM task group (TG) reports that are particularly relevant to HDR QA. They are TG 56: Code of practice for brachytherapy physics [3], and especially TG 59: HDR brachytherapy treatment delivery [4]. Another useful reference for brachytherapy quality assurance has been published by ESTRO and is available on their website [5]. This paper will provide details about HDR QA as it is performed in our radiation therapy department (on a Nucletron Microselectron system) and how the QA program is related to the NRC regulations and the task group recommendations.

## APPLICATOR QA

**Figure 1:**
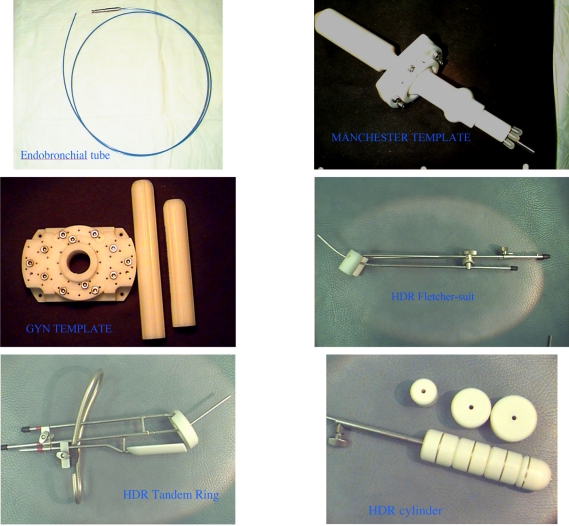
Examples of HDR applicators used for lung, rectal, and gynecologic diseases.

Prior to the initial use of a new (or replacement) applicator, it is necessary to verify that the source dwell positions correspond to the radiographic marker positions used in simulation and treatment planning. TG-56 recommends that coincidence of dummy and radioactive sources be checked annually as well. There are many standard applicators; photographs of several of those used in our institution are shown in Figure 1

**Figure 2:**
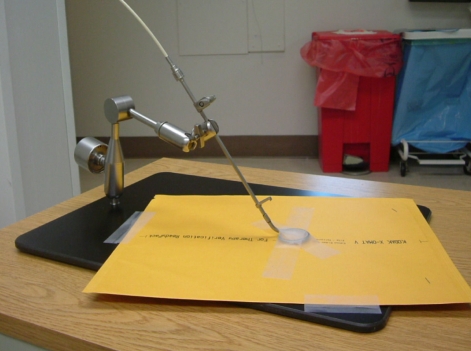
Photograph of a ring applicator secured to a base plate with film taped securely in place.

**Figure 3:**
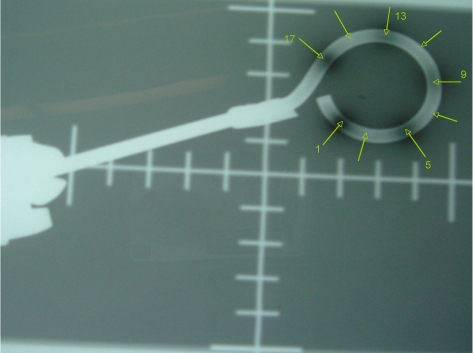
Autoradiograph of a 3 cm ring showing 5 dwell positions (1,5,9,13,17) as well as the intervening dummy markers (unnumbered arrows). Dwell positions are for the 0.5 cm step-size.

The method we employ to verify coincidence of dwell position and radiographic marker is autoradiography. An applicator is taped securely to a sealed film envelope (Figure 2) and the HDR after loader is programmed to send the source to a few appropriately chosen dwell positions for less than 1 second (e.g. 0.3 s for 0.31 GBq source). Next, the film plus applicator is transferred to a diagnostic X-ray source such as a simulator, the dummy source markers are placed in the applicator and the film exposed and developed (e.g. 125 kVp, 125 mAs for Kodak XV film). An example of this is shown in Figure 3. TG-56 recommends that the coincidence of dummy and active sources be within 2 mm; the NRC regulations call for ± 1 mm.

## PERIODIC SPOT-CHECK

The new NRC regulations require a periodic spot-check of each HDR unit prior to the first use on any given day that the after loader is in operation and after each new source installation. These spot-checks need not be done by the authorized medical physicist, but the latter must review the results and notify the licensee in writing of his findings. Table 1 lists the checks that must be performed at a minimum to assure proper operation of the unit according to NRC Regulations 10 CFR Part 35.

**Table 1:** Mandated periodic spot-checks.

1. Electrical interlocks at entrance to room
2. Source exposure indicator lights on the after loader, control console, and in the facility
3. Viewing and intercom systems
4. Emergency response equipment
5. Radiation monitors to indicate source position
6. Timer accuracy
7. Clock (date and time) in unit’s computer
8. Decayed source activity in unit’s computer

**Figure 4:**
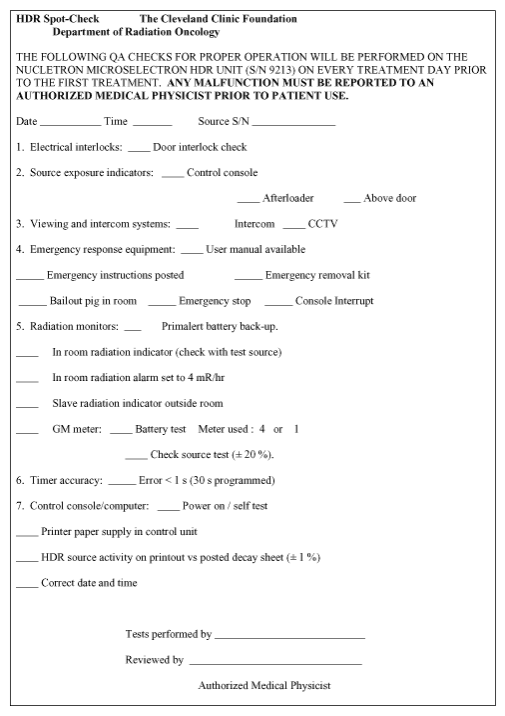
Spot-check form used each day of patient treatment. A downloadable version is available at http://www.biij.org/2006/2/e34/fig4.asp

A convenient way of implementing and recording the above quality assurance is by using a checklist such as the one our clinic uses as shown in figure 4

**Figure 5:**
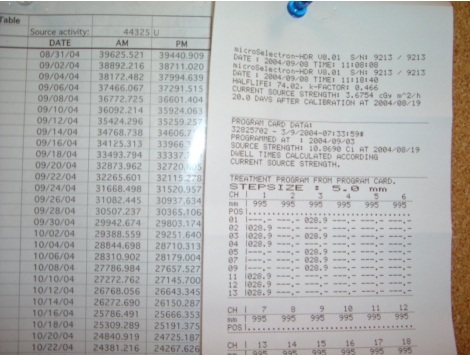
Source decay on physicist-generated spreadsheet (left) and printout from the HDR control console (right). The actual numbers for the particular treatment date are 36772 and 36754 mGy m2 h-1 (respectively).

Certain tests require only a simple inspection to ensure that materials are present, viz. User manual, Removal kit, Emergency instructions, Bailout pig, Radiation alarm setting, and Printer paper. Switching on the system allows the tests in item 7 to be performed. The source activity comparison can be made using a table generated by the medical physicist (Figure 5). This will also satisfy the requirement (see Full Calibration below) for performing decay correction which must be done by the authorized medical physicist. Agreement should easily be within 1 percent tolerances.

**Figure 6:**
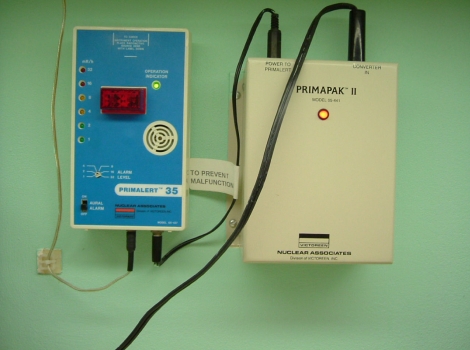
Exposure rate meter mounted on a wall in the treatment room so as to be visible from the entrance way.

For the remaining tests, the active source will need to be deployed. For this, the system can be programmed manually each time or a standard program recalled from the system memory. A single dwell time of 20 to 30 s suffices to test the door interlock, the interrupt button, and the emergency off button as well as to verify that the appropriate exposure indicators and radiation monitors are functioning properly. The spot check form requires testing of the functioning of the meter in the treatment room (Figure 6) under battery power alone. Its alarm setting of 4 mR/hr was established so as to be above exposure levels in the room due to an adjacent linac therapy suite. As an additional safety measure, we have a calibrated GM meter that is carried by hand by personnel upon entering the treatment room. It is checked using a 1 mCi Cs-137 source that yields a 10 mR/hr contact value. The remaining item is an estimate of timer accuracy. For this, a stopwatch is used to time a 30 s dwell. Typical error estimates are well below 1 second.

## FULL CALIBRATION

A “full calibration” is mandated for several different circumstances, e.g. before first medical use, following a source change or any major repair, etc. Since the source in most, if not all, modern HDR after loaders is Ir-192 with a half-life of approximately 74 days, the requirement for quarterly calibration [2] does not formally apply. However, it is usual to replace an iridium source four times a year so as to maintain reasonable dose rates and treatment times. Quarterly QA testing of HDR after loaders was recommended in the report of TG 56. The components of a full calibration as defined in NRC Regulations 10 CFR Part 35 are listed in Table 2

**Table 2:** Full calibration measurements (as applicable).

1. Output within ± 5%
2. Source positioning accuracy to within ± 1 mm
3. Source retraction with backup battery upon power failure
4. Length of the source transfer tubes
5. Timer accuracy and linearity over the typical range of use
6. Length of the applicators
7. Function of the source transfer tubes, applicators, and transfer tube-applicator interfaces

**Figure 7:**
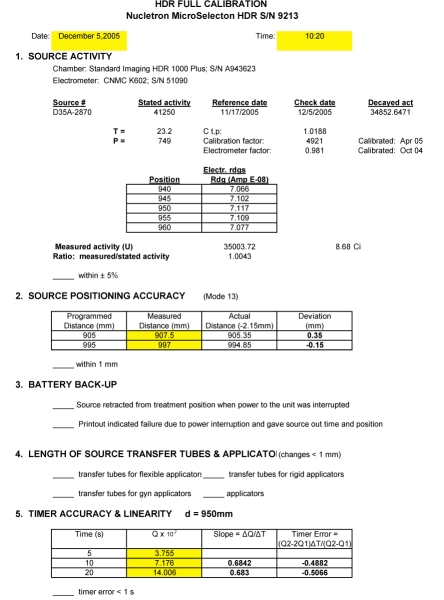
Full calibration spreadsheet with actual calibration data. A downloadable version is available at http://www.biij.org/2006/2/e34/fig7.asp

The form we employ for the full calibration is on an Excel spreadsheet (Figure 7) which allows convenient calculations of source activity and positioning as well as timer accuracy and linearity. The activity of the source is measured using a well chamber and electrometer having calibrations traceable to the National Institute of Standards and Technology (within 2 years as indicated by the dates on the form). The electrometer needs to be calibrated in both current and charge (integral) modes. The source is programmed to go to a series of positions within the well chamber and the maximum current reading is used to calculate the activity in air kerma units. This value is then compared to the manufacturer’s stated activity decayed to the day of measurement. Agreement is typically within 2%. The regulations allow a 5% range. If equipment calibrations traceable to a national standard are not available, dosimetry system constancy checks can be performed using a long-lived source such as Cs-137 [5]. This is not a desirable substitute for proper calibration.

**Figure 8:**
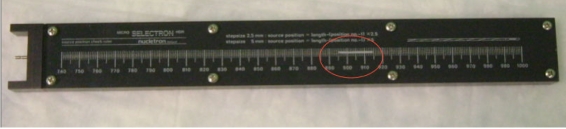
Source position ruler showing white plastic indicator (red circle).

**Figure 9:**
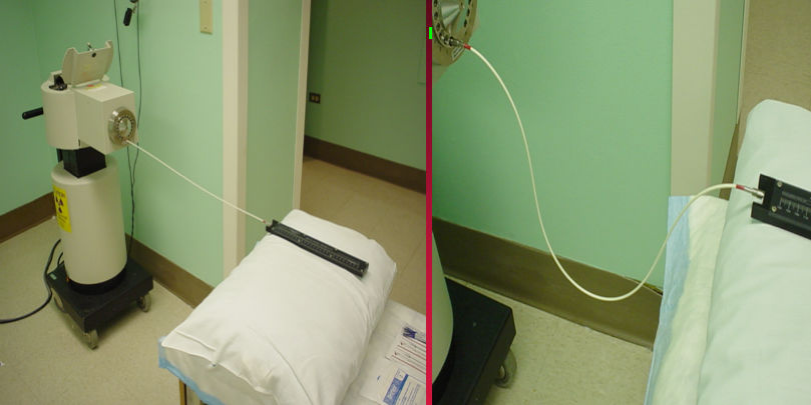
Source position accuracy test showing a well-aligned ruler, transfer tube, and afterloader (left) and a set-up with a large curvature.

Positioning accuracy is measured using a special ruler supplied by the manufacturer (Figure 8). The programmed position (e.g. 995) is for the center of the source, hence the correction (one-half of the source length, or 2.15 mm) for the leading edge. The one mm criterion may not be satisfied if there is much curvature in the measuring system (see Figure 9). Thus, some care must be taken to ensure that the transfer tube is reasonably straight and horizontal.

We perform the battery back-up test by shutting off the AC power to the after loader while a source has been deployed. This makes it a somewhat different test compared to the one in the spot-check where the emergency off button is pushed. That the source has been retracted is printed out at the control console and is verified by the radiation monitor indicating exposure rates below the set value (4 mR/hr).

Timer error and linearity are measured using a technique established for teletherapy sources. Charge is collected and measured in the well chamber for a set of predetermined times. The pass/fail criteria we adopted seem both reasonable and reproducible and well within the capability of the system.

We test the integrity of the transfer tube/applicator system in three ways.

- Once a program has been loaded into the control unit, a transfer tube + applicator is attached to Channel 1 but the indexer ring is not locked.
- The second test has the transfer tube removed from Channel 1 and the ring locked.
- The final test has the transfer tube inserted into Channel 1, the ring locked, and an applicator with an obstruction in it attached. It should be added that in the Nucletron system, failure to connect the transfer tube to the applicator properly will usually generate the same error code as an obstruction.

## TREATMENT PLANNING QUALITY ASSURANCE

It is standard practice in external beam radiotherapy to have a second, independent check of the treatment plan and monitor unit calculations. This may take the form of a simplified algorithm using data from phantom measurements or dose measurements inside a suitable phantom (especially for IMRT plans). For brachytherapy, the independent check is also desirable (but not mandated by NRC regulation), but there is no generally accepted method for doing it. Some characteristic parameter(s) of the plan must be compared to an expected value; however, what the characteristic parameters should be and how to arrive at the expected value are left to each medical physicist or institution. TG-59 addresses these issues and lists several approaches that have appeared in the literature [6-8]. Typically, the dose is calculated at representative points by the treatment planning system and then compared to the results from a second independent system (perhaps a spreadsheet or nomogram). It remains unclear what agreement is acceptable. Our method has been to use a plot of treatment time x source activity/ dose versus the global parameter of treatment volume for various applicator types. This is similar to the Paterson-Parker tables from the days of radium sources. It has been described previously [9] and will be summarised below.

The treatment volume (usually V100 in our experience) is obtained from the dose volume histogram (DVH). Several plans were run on both the Plato and a second treatment planning system and the respective DVH’s compared to lend credibility to the use of this parameter. We, then, used data from 20 to 30 patient plans for each of several applicator types (vaginal cylinder, tandem/ring, endobronchial tube) to construct the T*A/D vs V100 plots. Several cases for each applicator were double planned with a second treatment planning system (ROCS or Pinnacle) for verification purposes. The data on each graph were then fitted to either a straight line or a second order polynomial using statistical methods. The result was then used for checking new patient plans to ensure consistency. A summary of the initial use of this method for two types of applicators is shown in Table 3

**Table 3:** Percentage difference between newly created plans and our reference data.

**Applicator**	**linear** **average difference**	**polynomial** **average difference**	**n**
vaginal cylinder	5.45±.06%	2.76±.01%	34
tandem/ring	4.56±.02%	2.48±.02%	40

Clearly, the agreement is better when a polynomial is used for fitting the reference data. A similar situation is found for other types of applicators as well.

Perhaps an even more important aspect of treatment plan quality assurance is to have a second trained person inspect the plan and compare it with the written directive. The comparison should include such items as the dose prescription (per fraction and per course of treatment), the step size, dwell positions, etc. A more complete list is to be found in the report of TG-59. A check-list that is part of the patient’s chart is a practical method to ensure that this aspect of quality control is performed. Examination of other input data such as simulator films and comparison with the treatment plan is also essential. In our institution, specially trained radiation therapy technologists and the authorized user physician check the treatment plan.

## TRAINING OF PERSONNEL

The clinical personnel involved in an HDR program include the authorized user physician, authorized medical physicist, radiation safety officer, dosimetrist, nurse, and radiation therapy technologist. Some of these roles may be combined into one. For example, the medical physicist may act also as the radiation safety officer and do the treatment planning in lieu of a dosimetrist. The authorized physician and medical physicist should be certified by the appropriate medical specialties board and have had special training in brachytherapy. Of prime importance is the radiation safety training that all personnel involved in HDR treatments undergo. This is administered to new personnel and then annually for all those in the HDR program. Included is training in the proper response to a major emergency, in particular, failure of the source to be retracted into the after loader safe upon completion of treatment or upon power outage. The daily spot check should ensure that proper equipment (removal kit and bailout pig) is in place and that simple emergency instructions are posted so as to be readily available. If the source has to be retracted manually, the standard precepts of radiation safety, viz. time, distance, and shielding, should be followed. If operation of the hand crank is unsuccessful, then the applicator containing the stuck source has to be removed from the patient. Once again, speed is crucial as is having such items as long forceps and a flashlight on hand. With the applicator plus source placed in the bailout pig and the patient and medical personnel removed from the treatment room, the HDR suite should be secured and a service engineer contacted for repair. We find it useful at the time of the annual training to review and discuss in detail what each member of our brachytherapy team would do in various emergency situations.
